# Supplementary material for: Patterns of Intron Gain and Loss in Fungi
Source: PLoS Biol. 2004 Nov 30;2(12):e422. doi: 10.1371/journal.pbio.0020422 (PMC532390; doi:10.1371/journal.pbio.0020422)
Supplement: Table S1 — Also available at http://genes.mit.edu/NielsenEtAl/. (4.3 MB ZIP). [file pbio.0020422.st001.zip › NielsenEtAl/html/137.html]

AN7146.1.NCU03006.1.MG10568.1.FG02783.1


```
 CLUSTAL W (1.82) Multiple Sequence Alignments - Introns Inserted


Sequence 1: AN7146.1	377 aa
Sequence 2: FG02783.1	381 aa
Sequence 3: NCU03006.1	379 aa
Sequence 4: MG10568.1	380 aa
Alignment Length: 381 aa
Number Identitical Residues: 231 aa
Alignment Score (without introns) 10757


MG10568.1 	-MGAPKQGLEKEDKKRDAEFNKALHGQSALAAGGIAAMFSKDKEAKKLAVDEYFKHFDNK
NCU03006.1	-MPS-TIALEKEDHQRDAEFKKVMHGDSAKAAGGVAALLKKNTEAQQIAVDEYFKHFDNK
FG02783.1 	MVASSNTGLEREDHQRDADFNKAMHGSSAQARGGVAAMFRKGGAAKQAAVDEYFKHWDNK
AN7146.1  	---MAPTALEKENHQRDAEFNAAMHGNSAQAKGGFAAMRQKSAAAQKAAVDEYFKHWDNK
          	    .  .**:*:::***:*: .:**.** * **.**:  *.  *:: ********:***

MG10568.1 	TAANETPEERA0ARQKEYATLTRH~YYNLATDIYEYGWGQSFHFCRFSHNESFYQAIARH
NCU03006.1	TAEAETQADRE~ARTKEYATLTRH2YYNLATDIYEYGWGQCFHFCRYSPGESFYQAIARH
FG02783.1 	PAENETPEERA0ARQAEYATLTRH2YYNLATDLYEYGWGQSFHFCRFSQGEPFYQAIARH
AN7146.1  	SAETETEEIRE0ARRAEYATLTRH2YYNLATDLYEYGWGTSFHFCRFAYGEPFYQAIARH
          	.*  **   *  **  ******** *******:****** .*****:: .*.********

MG10568.1 	EHYLAHQIGIKEGMTVLDVGCGVGGPAREIAKFTGCNVVGLNNNDYQIDRATHYAKQEKL
NCU03006.1	EHYLAAQIGIKKDMKVLDVGCGVGGPAREIAKFTDAHITGLNNNDYQIDRATHYAVRDGL
FG02783.1 	EHYLAHQIGIKDGMKVLDVGCGVGGPAREIAKFTGAHITGLNNNNYQIERATHYAFKEGL
AN7146.1  	EHYLAHQMGIKPGMKVLDVGCGVGGPAREIVKFTDAHVTGLNNNDYQIQRATRYAEREGL
          	***** *:*** .*.***************.***..::.*****:***:***:** :: *

MG10568.1 	DSQLQFVKGDFM0QMSFPDESFDAVYAIEATVHAPKLEGVYSEIFRVLKPGGTFGVYEWL
NCU03006.1	SGQLKFVKGDFM~QMSFPDNSFDAVYAIEATVHAPKLEGVYGEIYRVLKPGGTFGVYEWL
FG02783.1 	SNQLEFVKGDFM0QMSFPDNSFDAVYAIEATVHAPTLKGIYSEIFRVLKPGGVFGVYEWL
AN7146.1  	SHKLNFVKGDFM~QMQFPDNSFDAVYAIEATVHAPELAGVYKEIMRVLKPGGVFGVYEWL
          	. :*:******* **.***:*************** * *:* ** *******.*******

MG10568.1 	MTDDYDNDNLHHRDIRLKIELGNGISNMVPISEGEAAMRAAGFEIIKTDDLAQKGDELPW
NCU03006.1	MTDNYDNDNVEHRDIRLAIEVGNGISNMVTISEGLAAMKNVGFELVHHEDLADRNDPMPW
FG02783.1 	MTDEYDNDNLRHREIRLGIEQGDGISNMCKVSEGIAAIHDSGFEMLHHEDLADRPDALPW
AN7146.1  	MTDAYDNDNPEHRKIRLGIEQGDGISNMVKVSEGLAAIKEAGLELLHHEDLADRPDEIPW
          	*** ***** .**.*** ** *:*****  :*** **::  *:*::: :***:: * :**

MG10568.1 	YWPLAGDLKYLQSYFDLFTVLRMTKWARTCLHGMAGVLEAVGVAPAGTKKTADALATGGD
NCU03006.1	YWPIAGELRYMQSYLDFFTVVRMTHTARRILHGFAGILETVGLAPKGTKKTADALARGAD
FG02783.1 	YWPLAGELRYVQTVGDFFTIVRMTTWGRTIAHGLAGLLETFKLAPAGTKKTADSLALAAD
AN7146.1  	YYPLAGSFKHMTSPWDFFTIARMTWWGRGLVHRFVGAMETIKLIPQGTQKTADSLALAAD
          	*:*:**.:::: :  *:**: ***  .*   * :.* :*:. : * **:****:** ..*

MG10568.1 	ALVAGAKENIFTPMYLMVGRKPMN
NCU03006.1	GLVAGAKKKLFTPMYLMVGKKPLN
FG02783.1 	CLVAGGRDKLFTPMYLMVARKPAA
AN7146.1  	CLVAGGEKKLFTPMYLMVARKPE-
          	 ****...::********.:**
```
